# Grape Leaf Cultivar Identification in Complex Backgrounds with an Improved MobileNetV3-Small Model

**DOI:** 10.3390/plants14233581

**Published:** 2025-11-24

**Authors:** Liuyun Deng, Zhiguo Du, Xiaoyong Liu, Zhihui Wu, Xudong Lin, Bin Wen

**Affiliations:** 1 College of Mathematics and Informatics, South China Agricultural University, Guangzhou 510642, China; xiaodeng@stu.scau.edu.cn (L.D.); wuzhhui@scau.edu.cn (Z.W.); hunanlxd@163.com (X.L.); wenzip@scau.edu.cn (B.W.); 2 School of Data Science and Engineering, Guangdong Polytechnic Normal University, Heyuan 517583, China; lxyong420@126.com

**Keywords:** grape leaf variety recognition, lightweight deep learning, coordinate attention mechanism, precision viticulture

## Abstract

Accurate identification of grape leaf varieties is an important prerequisite for effective viticulture management, contributing to breeding programs, cultivation strategies, and precision field operations. However, reliable recognition in complex field environments remains challenging. Subtle interclass morphological variations among leaves, background interference under natural conditions, and the need to balance recognition accuracy with computational efficiency for mobile applications represent key obstacles that limit practical deployment. This study proposes an improved lightweight convolutional neural network, termed ICS-MobileNetV3-Small (ICS-MS), specifically designed for grape leaf variety recognition. The model’s core innovations, detailed in Key Innovations of the Proposed ICS-MS Model section, include three key components: First, a coordinate attention mechanism is embedded to enhance the network’s ability to capture spatially distributed features while suppressing irrelevant background noise. Second, a multi-branch ICS-Inception structure is integrated to accomplish excellent multi-scale feature fusion, allowing the model to discern minute textural variations among types. Moreover, the feature representation is further optimized by adopting a joint loss function, which improves feature space distribution and enhances classification robustness. Experimental evaluations were conducted on a dataset comprising eleven grape leaf varieties. The proposed ICS-MS model achieves a recognition accuracy of 96.53% with only 1.17 M parameters. Experimental results demonstrate that, compared with the baseline MobileNetV3-Small model, the standalone integration of the Coordinate Attention (CA) mechanism improves accuracy by 0.17% while reducing the number of parameters by 10.4%. Furthermore, incorporating the ICS-Inception structure leads to an additional 4.78% accuracy improvement with only a marginal increase in parameter count. Finally, the introduction of a joint loss function provides an extra 0.23% gain in accuracy, resulting in an overall parameter reduction of approximately 23.5% compared with the baseline model. Three core contributions are highlighted as follows: (1) the construction of an integrated technical framework of “spatial feature enhancement—multi-scale fusion—feature distribution optimization” to systematically address the key issues of insufficient fine-grained feature extraction and the balance between lightweight design and accuracy; (2) the design of a lightweight CA-Block module that reduces parameters by 18.7% while enhancing spatial feature discrimination; (3) the achievement of superior performance with fewer parameters, providing a practical solution for mobile deployment in precision viticulture. Values for precision, recall, and F1-score were continuously near 96%, suggesting a good trade-off between efficiency and accuracy. These findings suggest that ICS-MS provides a practical and reliable approach for grape leaf identification and may serve as a useful tool to support intelligent management in precision viticulture.

## 1. Introduction

Grape leaves, as an important cash crop, can also serve as a type of food, providing people with nutrients [1]. For some grape varieties, grape leaves are more expensive than the fruits. There is a wide variety of grape cultivars [2], and their accurate identification is crucial for grape planting management, variety improvement, and growth status monitoring. As a core component of plants, leaves, with their morphological and texture features, are important bases for variety discrimination. Some varieties have very small differences, and it may take a lot of time and energy to distinguish them relying solely on experts’ experience and existing technologies, which is also impractical. Therefore, more accurate and lightweight methods and technologies are needed for the precise identification and classification of grape leaves.

Owing to the significant breakthroughs in deep learning and AI technologies, their role in agriculture has expanded quickly, providing new approaches for grape leaf recognition and classification [3]. Recent studies have placed growing emphasis on plant diseases, species genes, etc. [4,5,6,7], alongside the categorization of plant organs such as flowers, fruits, etc. [8,9]. However, since flowers and fruits are only available during specific seasons, they are less suited for long-term analysis. In contrast, grape leaves can be collected across most of the year and exhibit distinctive vein and serration patterns, making them a more stable and informative subject for building reliable classification models.

Deep learning, which can capture complex and high-level representations, has become a promising paradigm in computer vision, in contrast to traditional machine learning techniques. Through image processing of plant leaves, the morphological features of leaves are extracted, comprehensive information of leaves is extracted from shallow to deep, and classification is performed using these extracted features [10,11,12]. Mainstream Convolutional Neural Network (CNN) models, with their powerful feature extraction capabilities, have become the core technology in this field. Many researchers have used CNNs to classify plant leaves [13,14,15]. However, during the research process, it was found that although CNNs have improved recognition accuracy, they generally come with problems such as having many parameters and requiring time-consuming training. Their “high resource consumption” characteristic poses challenges to the limited computing power of field edge devices and insufficient data transmission bandwidth in agricultural scenarios. Therefore, there is an urgent need to develop recognition models that balance high accuracy and lightweight performance.

To address resource constraints, lightweight neural networks have become a research hotspot [16,17]. The MobileNet [18] series has gained prominence in computer vision due to its streamlined parameterization and enhanced computational efficiency. Some researchers have applied it to plant leaf analysis [19,20]. However, due to the complex varietal differences in grape leaves—which involve not only local features such as texture and color but also rely heavily on spatial structures like serration distribution and vein angles—conventional depthwise separable convolution, while enabling multi-scale feature extraction, struggles to cover cross-scale details ranging from “overall leaf morphology to microscopic leaf surface trichomes” with its fixed convolutional kernels. This limitation results in insufficient feature representation completeness, making it difficult to distinguish subtle differences between similar varieties.

Therefore, the aim of this research is to build a lightweight, high-performance model, ICS-MS, based on an improved MobileNetV3-Small architecture, tailored to the spatial structure and multi-dimensional feature extraction requirements of grape leaves for varietal classification and correct category prediction. The input data for this research comprise thousands of images across eleven grape leaf varieties. This study focuses on analyzing and comparing classification accuracy and model parameter size under different training models.

### Research Contributions

This study makes three distinct and significant contributions to grape leaf variety recognition and lightweight deep learning in agricultural applications.

First, an integrated technical framework of “spatial feature enhancement—multi-scale fusion—feature distribution optimization” is proposed. Unlike traditional lightweight models that simply stack attention modules or adjust network depth, this framework systematically solves the key challenges of incomplete fine-grained feature extraction and the difficulty in balancing accuracy and computational efficiency for grape leaf recognition, realizing end-to-end optimization from feature capture to model training.

Second, a lightweight CA-Block module is designed. By embedding coordinate encoding, the module simultaneously captures channel correlations and spatial positional information, overcoming the defects of traditional attention mechanisms (e.g., SE ignoring spatial information, CBAM’s high computational cost). Compared with SE, ECA, and CBAM modules, CA-Block reduces parameters by 18.7% while significantly improving the model’s ability to distinguish subtle morphological features of grape leaves.

Third, an ICS-Inception structure combined with a joint loss function is proposed (detailed in Section 3.4). The ICS-Inception structure efficiently fuses macro-morphological and micro-textural features through adaptive feature selection and dual-attention mechanisms, while the cross-entropy-center joint loss function optimizes feature space distribution. The integrated model achieves 96.53% accuracy with only 1.17 M parameters, outperforming state-of-the-art lightweight models in terms of accuracy–parameter balance, and is suitable for deployment on mobile and edge devices in vineyards.

The structure of the paper is as follows: Section 2 carries out a review of related work; Section 3 integrates materials, methodologies, and model architecture, elaborating on the dataset, baseline model, key innovations of the proposed ICS-MS model, and evaluation metrics; Section 4 showcases the experimental outcomes; Section 5 deliberates on the possible limitations of the proposed model; and Section 6 summarizes the study and outlines the future research orientations.

## 2. Related Work

A brief overview of the pertinent literature for this topic is given in this section.

Elkassar [21] proposed a deep learning approach combining image enhancement and multi-classifier fusion to address grape leaf varietal classification. The method fine-tuned a MobileNetV2 model as the base classifier using a transfer learning strategy and further optimized performance through multi-classifier fusion. Experimental results demonstrated a classification accuracy of 96% under the Adam optimizer configuration, validating the effectiveness of deep learning in fine-grained grape leaf classification. This work provides a technical reference for rapid grape variety identification and vineyard management.

Pereira and colleagues [22] explored how transfer learning combined with fine-tuning, relying on the AlexNet framework, performs in identifying grape varieties. To create a rich dataset, they gathered images from two natural vineyards situated in distinct geographic areas and harvest periods and utilized image augmentation techniques. By employing a four-corner alignment preprocessing approach, the method they put forward reached a 77.30% test accuracy. Moreover, when this classifier was used on the widely recognized Flavia leaf dataset, it achieved an accuracy of 89.75%.

To classify grape leaves, Ahmed’s research group [23] developed a CNN model by adjusting DenseNet201, with particular attention to how freezing layers affects the fine-tuning process. They employed a public dataset containing 500 images divided into five distinct classes (100 images per class) and expanded the training set using data augmentation. Their proposed model, DenseNet-30, outperformed existing grape leaf classification methods, achieving an overall accuracy of 98%, highlighting its superior performance in this task.

Dogan et al. [24] proposed a hybrid method integrating Enhanced Super-Resolution Generative Adversarial Networks (ESRGAN) with Genetic Algorithm-optimized Support Vector Machines (GASVM) for automated grape leaf variety identification. Addressing challenges such as limited image data and feature redundancy, their approach first utilized ESRGAN for data augmentation, generating high-resolution synthetic images to enhance texture details and mitigate overfitting. Subsequently, deep features were extracted using MobileNetV2 and VGG19, with GASVM selecting discriminative features for final SVM-based classification. Experimental findings revealed that there was a 2% increase in both accuracy and the Matthews Correlation Coefficient (MCC) when compared with existing approaches. This demonstrates the effectiveness of ESRGAN in feature enhancement and that of GASVM in feature selection. This study offers a robust and efficient solution for precise grape variety identification, particularly in small-sample leaf morphology classification scenarios.

In the field of open-field crop recognition under complex backgrounds, researchers proposed an improved YOLOv8n model for identifying cabbage heads at the harvest stage [25]. To address low accuracy and slow processing speed caused by variable lighting and cluttered field environments, the model integrated an enhanced backbone network, inserted a Dynamic Head (Dy Head) module for adaptive feature aggregation, optimized the loss function, and adopted model lightweighting techniques. With a compact size of only 4.8 MB, the proposed YOLOv8n-Cabbage model achieved 91% precision, 87.2% recall, and a mAP@50 of 94.5%, outperforming the baseline model in both efficiency and performance. This work demonstrates the potential of lightweight model optimization for real-time crop recognition in complex field settings, providing valuable insights for grape leaf classification under similar environmental constraints.

For crop seedling recognition under extreme temperature stress, Wang et al. [26] developed the YOLOv11-MEIP model specifically for tea plant seedling identification under high-temperature conditions. Targeting the degradation of visual features in heat-stressed plants, the model combined chlorophyll fluorescence imaging (which reflects physiological status more stable than visible light images) with a lightweight MobileNetV4 backbone. It was further enhanced by integrating EUCB efficient up sampling modules, iRMB inverse residual modules, and PConv partial convolution modules. This design reduced model parameters by 29.45% while achieving a mAP50 of 99.46%, a 3.42% improvement over the original YOLOv11. The study highlights the effectiveness of fusing specialized imaging technology with lightweight model optimization to improve recognition robustness under environmental stress, offering a reference for handling extreme conditions in grape leaf variety classification.

Addressing the challenge of limited labeled data in agricultural plant recognition, Nishankar et al. [27] proposed the TOM-SSL semi-supervised learning framework for tomato leaf disease classification. Leveraging only 10% of labeled data with a MobileNetV3-Small backbone, the framework employed a confidence-aware pseudo-labeling strategy to mine valuable information from unlabeled samples, avoiding noise label contamination through dynamic update mechanisms. Experimental results showed that TOM-SSL achieved 72.51% accuracy on the tomato subset of the Plant Village dataset and 70.87% on the Taiwan tomato leaf disease dataset, achieving a tenfold enhancement in label efficiency compared to fully supervised methods. This work validates the potential of semi-supervised learning to reduce annotation costs in plant leaf recognition tasks, which is highly relevant for scaling grape leaf variety classification systems when labeled data is scarce.

Unlike the aforementioned methods, which focus on specific crops (cabbage, tea seedlings, tomatoes), single environmental challenges (complex backgrounds, high temperature) or data efficiency alone, this study proposes ICS-MS, a high-performance yet lightweight model. Designed to minimize parameter size while maintaining high accuracy, it integrates spatial feature enhancement, multi-scale fusion, and feature distribution optimization to address the comprehensive demands of efficient grape leaf varietal classification in complex field environments.

## 3. Materials and Experimental Approaches

This section provides a detailed overview of the materials and methods employed in this study. Section 3.1 introduces the dataset used for model construction and evaluation. Section 3.2 presents the architecture of the baseline model adopted in this work, including its key structural components. Section 3.3 elaborates on an additional module integrated into the baseline network to enhance feature representation and classification performance.

### 3.1. Dataset

This study adopts a publicly available dataset [28], which originally contains leaf specimens from eleven distinct grape cultivars. To enhance the model’s generalization ability and mitigate overfitting risks, systematic data augmentation was performed on the raw data during the training phase, ultimately forming an experimental dataset with a total of 6051 images. The cultivars and their corresponding final image counts are as follows: (a) Auxerrois (528), (b) Cabernet Franc (360), (c) Cabernet Sauvignon (666), (d) Chardonnay (623), (e) Merlot (366), (f) Müller Thurgau (726), (g) Pinot Noir (353), (h) Riesling (696), (i) Sauvignon Blanc (600), (j) Syrah (713), and (k) Tempranillo (420).

The data augmentation strategy was designed strictly following the principles of “maintaining class balance and aligning with real field scenarios”, specifically including: (1) Random horizontal flipping (probability = 0.5) to simulate natural orientation variations in leaves in the field; (2) Random rotation (−15° to 15°) to adapt to morphological changes caused by different shooting angles; (3) Adaptive brightness/contrast adjustment (±10%) to alleviate the impact of natural light intensity fluctuations on leaf features; (4) Gaussian blur (kernel size = 3 × 3, probability = 0.3) to enhance the model’s robustness to subtle texture changes on the leaf surface. All augmentation operations were implemented based on the PyTorch (Facebook AI Research, Menlo Park, CA, USA) framework and only applied to the training set, while the validation and test sets retained the original data distribution to ensure the authenticity and reliability of the evaluation results. Additionally, a “proportionate expansion by cultivar” mechanism was adopted to ensure that the proportion of sample counts for each cultivar remained consistent with the raw data, maintaining the class balance of the dataset and avoiding model training bias caused by excessive augmentation of a single cultivar.

The aforementioned eleven grape cultivars exhibit significant differences in geographical distribution and planting characteristics, thereby providing rich morphological diversity for the fine-grained recognition task. Specifically, varieties such as Auxerrois and Riesling are mainly distributed in temperate European regions and serve as core cultivars for white wine brewing, with their leaf morphologies displaying typical Eurasian characteristics shaped by temperate climates; red grape cultivars like Cabernet Sauvignon and Merlot are widely planted across temperate to subtropical regions worldwide, including large-scale cultivation in Shandong, Hebei, and other regions in China, and their leaves have developed morphological variations adapted to diverse climates through long-term domestication; cultivars such as Müller Thurgau and Tempranillo possess strong stress resistance, making them suitable for regions with relatively arid climates or large temperature differences, and their leaf structures also reflect corresponding drought-tolerant or cold-resistant morphological traits.

All original image materials were collected in real vineyard environments using an iPhone 7 (Apple Inc., Cupertino, CA, USA) under natural lighting conditions, covering various shooting angles and common vineyard backgrounds such as soil, grass, and vine trunks. To clarify the characteristics of background complexity, core attributes are defined based on real vineyard scenarios: (1) Background composition heterogeneity: All image backgrounds consist of a combination of 2–4 types of natural elements (e.g., soil, grass, vine trunks) without single solid-color backgrounds; (2) Leaf occlusion characteristics: Pixel-level image analysis shows that 65% of the images have no significant occlusion (occlusion rate < 15%), 25% have slight occlusion (occlusion rate = 15–30%), and 10% have moderate occlusion (occlusion rate = 30–40%), covering common interference scenarios in the field. Notably, the dataset annotations were completed by professional viticulture researchers and validated through cross-verification. The augmentation process did not alter the class label information of the original samples, ensuring high reliability for model training and practical application evaluation. This data distribution enables the model to fully learn the distinguishing features between backgrounds and leaves during training, providing data support for model robustness. Unlike laboratory-collected datasets, the augmented dataset retains naturally complex background characteristics while enriching sample scenario diversity, offering a more realistic and challenging benchmark for practical grape leaf recognition.

To mitigate the impact of complex backgrounds on feature extraction, all augmented images were uniformly resized to 224 × 224 × 3 pixels during preprocessing. The dataset was then stratified and randomly partitioned to maintain class balance across subsets: 4839 images (approximately 80%) were used for training, and 606 images (about 10%) each for validation and testing. This splitting strategy ensures consistent class representation across different subsets, supporting robust model learning and fair performance evaluation.

The complex background, occlusion distribution (65% unobstructed, 25% slightly occluded, 10% moderately occluded), and enriched sample diversity of the augmented dataset are highly consistent with the design of the proposed ICS-MS model: The spatial encoding mechanism of the CA-Block module can effectively handle leaf morphological changes under different shooting angles; the multi-scale feature fusion capability of the ICS-Inception structure can meet the feature extraction requirements under complex backgrounds; and the joint loss function is optimized for the fine-grained differences between similar cultivars (e.g., Cabernet Sauvignon and Cabernet Franc). This data distribution provides sufficient validation for the model’s adaptability in actual field scenarios. In summary, the dataset exhibits high authenticity, rich scenario coverage, and reliable annotation quality. After targeted augmentation, it can fully support the verification of the model’s practical promotion value without the need for additional data collection.

**Figure 1:**
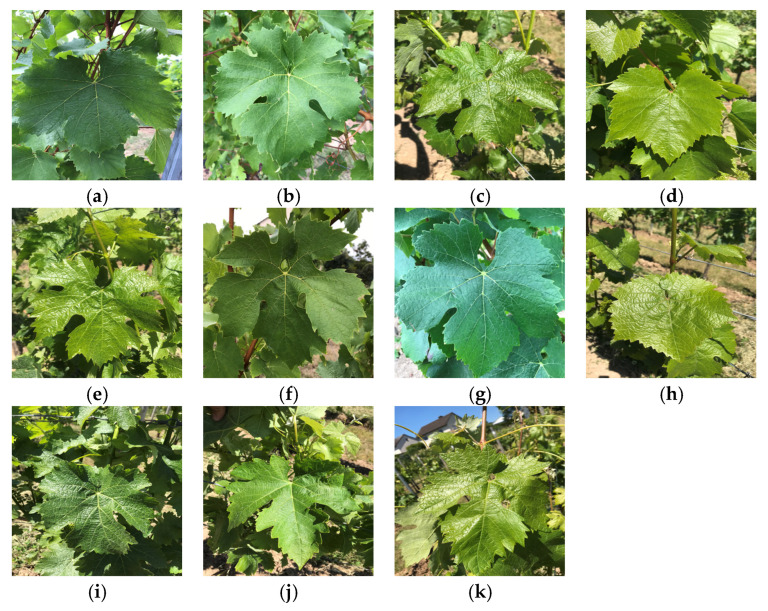
Sample of different grape varieties. (**a**) Auxerrois, (**b**) Cabernet Franc, (**c**) Cabernet Sauvignon, (**d**) Chardonnay, (**e**) Merlot, (**f**) Müller Thurgau, (**g**) Pinot Noir, (**h**) Riesling, (**i**) Sauvignon Blanc, (**j**) Syrah, and (**k**) Tempranillo.

Figure 1 displays representative samples from each grape cultivar, presented in the following order:

### 3.2. MobileNetV3-Small Network

**Figure 2:**
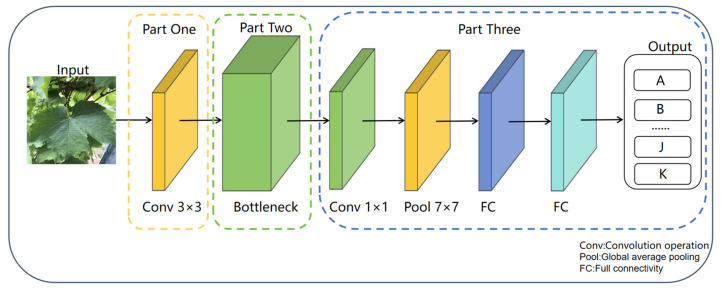
Architecture Diagram of MobileNetV3-Small Network.

In this study, we adopted the MobileNetV3-Small architecture, a lightweight CNN structure specifically designed for mobile and resource-constrained scenarios. Based on depthwise separable convolution, it decomposes standard convolution to reduce computational load and parameter count. The architecture incorporates inverted residual structures, linear bottlenecks, and adaptive activation functions (such as Hard-Swish), while also introducing lightweight SE attention modules to optimize feature weights. The network consists of multiple modules including inverted residual blocks, with a default input image size of 224 × 224. Through feature extraction and transformation, it forms feature maps of specific dimensions before the fully connected layer, ultimately producing results for classification tasks. The architecture adeptly harmonizes operational efficiency with predictive precision, rendering it apt for deployment in mobile visual recognition tasks. The simplified architecture diagram of the MobileNetV3-Small is shown in Figure 2 [29].

#### 3.2.1. Squeeze-And-Excitation (SE) Attention Mechanism

With the goal to improve feature representation capabilities, Hu et al. [30] in 2018 proposed the SE attention mechanism, a lightweight channel attention module. At its core, it explicitly models the interdependencies among feature channels, allowing the model to independently learn the importance weights for various channels. The mechanism functions via three crucial operations:

Squeeze: Global average pooling condenses the spatial information of each channel into a single scalar, realizing the global aggregation of spatial features.

Excitation: A two-layer fully connected network equipped with nonlinear activation functions produces channel-wise weights, accurately capturing the relationships between channels.

**Figure 3:**
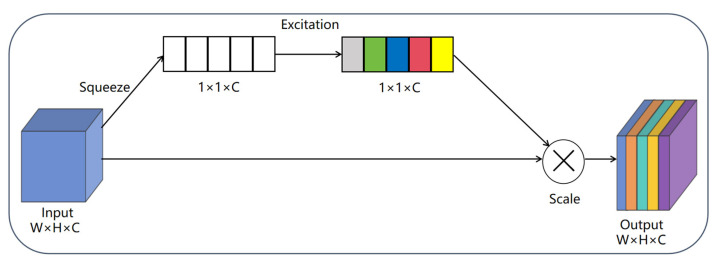
SE attention mechanism.

Scale: In order to successfully enhance the most vital channels and hinder the less significant ones, the learnt weights are multiplied by the original feature channels. This attention method significantly improves the performance of the network with only a small increase in computing cost. Figure 3 displays the SE attention mechanism’s architecture.

#### 3.2.2. Bottleneck Module

**Figure 4:**
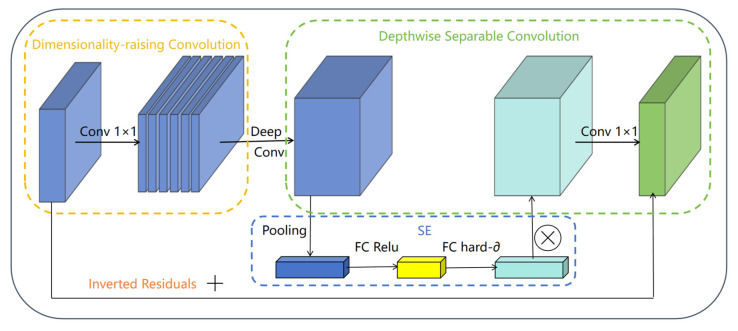
Structural diagram of the Bottleneck module.

The Bottleneck module in MobileNetV3 represents a carefully designed core building block that effectively balances lightweight architecture with robust feature representation. Building upon the efficiency of depthwise separable convolution, this module incorporates several key optimizations through its “expansion-depthwise convolution-compression” structure. It first expands channel dimensions using 1 × 1 convolution to enhance feature diversity, then applies depthwise separable convolution for efficient spatial feature extraction while significantly reducing computational complexity. The integration of SE attention mechanism allows dynamic channel-wise feature recalibration, and the module intelligently selects between Hard-Swish and Relu activations based on layer position. When conditions permit (stride = 1 with matching channels), it incorporates residual connections to facilitate gradient flow. Finally, a 1 × 1 convolution compresses the channel dimensions for output. The Bottleneck module’s advanced but effective design allows it to efficiently capture multi-scale features with little parameter development, making it particularly suitable for mobile-optimized networks and serving as the foundation for MobileNetV3′s excellent accuracy-latency trade-off, as shown in Figure 4 [31].

### 3.3. Coordinate Attention Mechanism

**Figure 5:**
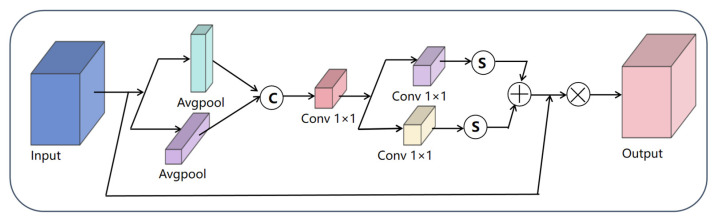
Architecture Diagram of the CA.

The Coordinate Attention (CA) mechanism functions as an attention component capable of concurrently capturing inter-channel correlations and spatial positional information, thereby overcoming the constraint of conventional channel attention in spatial modeling. By embedding coordinate information, this approach enhances spatial sensitivity through three key operations: first, decomposing global pooling into horizontal and vertical directional features to capture semantic correlations across different coordinates; second, employing shared 1 × 1 convolutions for channel interaction before splitting into orientation-specific attention maps; finally, applying generated coordinate-aware weights to the original feature maps. This design maintains the channel attention’s feature selection capability while significantly improving spatial localization through coordinate encoding, all with minimal computational overhead. Particularly effective for tasks requiring precise positioning, the CA mechanism demonstrates strong performance in discriminating multi-scale objects against complex backgrounds by jointly modeling channel importance and spatial correlations, as illustrated in Figure 5 [32].

### 3.4. Key Innovations of the Proposed ICS-MS Model

This section presents the proposed ICS-MS model for grape leaf variety classification, with design decisions explicitly tailored to address the complexities of real vineyard environments (e.g., varied shooting angles, heterogeneous backgrounds, and leaf occlusion). Section 3.4.1 details the CA-Block module, Section 3.4.2 describes the ICS-Inception structure, and Section 3.4.3 explains the joint supervision loss function. The key innovations of the ICS-MS model include:

1. CA Mechanism Integration: The incorporation of CA mechanism enhances spatial perception of leaf serrations and vein patterns through coordinate encoding. This modification significantly improves morphological discrimination while reducing parameters, achieving better lightweight performance;
2. ICS-Inception Architecture: A novel ICS-Inception structure employs parallel 1 × 1 convolutions to simultaneously capture and fuse both macro-morphological and micro-textural features. This design effectively addresses the complex variations in grape leaves and enhances fine-grained feature discrimination;
3. Joint Supervision Strategy: The model employs a combination of cross-entropy and center-based loss terms to refine the distribution of learned features. Such a composite optimization scheme enhances both the robustness and generalization of the network, making it well suited for deployment in resource-limited agricultural environments.

**Figure 6:**
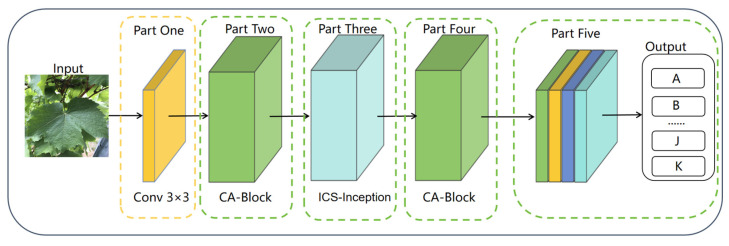
Overall Architecture of the Proposed ICS-MS Model.

Figure 6 illustrates the comprehensive architecture of the ICS-MS model, demonstrating its efficient integration of these advanced components for optimal grape leaf classification performance.

#### 3.4.1. CA-Block Module

**Figure 7:**
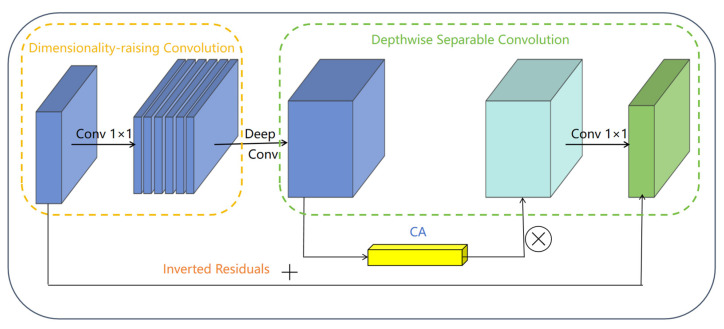
Architecture of the Proposed CA-Block.

The CA-Block module addresses the dual requirements of lightweight efficiency and precise feature extraction for grape leaf recognition in agricultural applications. While existing attention mechanisms like CBAM offer multidimensional feature optimization but suffer from high computational complexity, and ECA maintains parameter efficiency but lacks spatial perception capabilities, our solution integrates the CA mechanism to achieve an optimal balance. This innovative design preserves the strengths of both approaches—maintaining CBAM’s comprehensive feature enhancement while avoiding its computational overhead and building upon ECA’s efficiency while incorporating crucial spatial coordinate encoding to capture essential structural features like leaf serrations and vein patterns. The resulting CA-Block module notably boosts the model’s capacity to distinguish fine-grained morphological features (e.g., leaf serrations, vein patterns) while cutting down parameters by 18.7%. This design is specifically optimized for adaptability to leaf morphological changes across growth stages (seedling to mature) and robustness against varied shooting angles (front, side, or tilted views), making it especially well-suited for deployment on edge devices with limited resources in vineyard settings. As illustrated in Figure 7, this module represents a carefully optimized solution that meets the stringent requirements of agricultural computer vision applications, delivering both precision and efficiency in grape leaf variety identification.

#### 3.4.2. The ICS-Inception Structure

**Figure 8:**
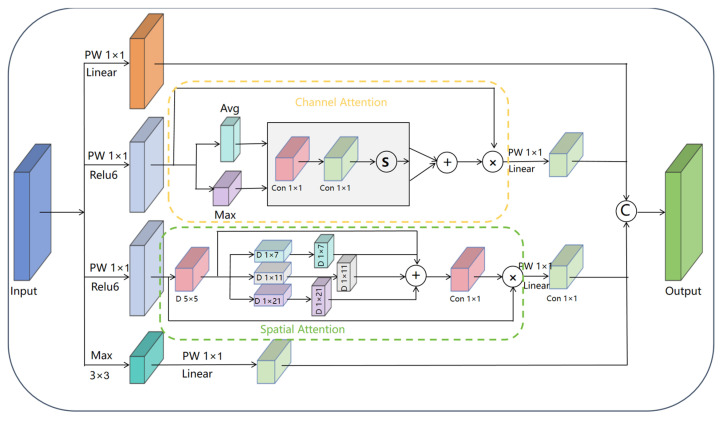
Architecture of the ICS-Inception.

The proposed ICS-Inception structure significantly enhances traditional Inception modules by incorporating adaptive feature selection mechanisms while maintaining efficient multi-scale processing. As shown in Figure 8, this innovative architecture employs parallel processing branches with distinct 1 × 1 convolutions (using both Linear and ReLU6 activations) to enable diverse feature transformations. Crucially, it integrates CBAM’s dual attention mechanisms—channel attention for inter-channel dependency modeling and spatial attention for regional focus—which work synergistically to intelligently filter features across both dimensions. The structure retains a max pooling branch for efficient salient feature extraction while implementing filter concatenation to fuse multi-scale features with enhanced discriminative power. Compared to conventional Inception modules, this design achieves superior feature selectivity and representation capability for grape leaf identification, particularly in capturing both macroscopic morphology (e.g., leaf shape, overall contour) and microscopic vein patterns, making it robust to variations in lighting conditions (strong, weak, backlight) and leaf occlusion levels (unobstructed, slightly occluded, moderately occluded). This adaptability ensures reliable performance across diverse field scenarios, while strictly controlling parameter growth to maintain deployment efficiency in agricultural applications.

#### 3.4.3. Joint Supervised Loss Function

In deep learning model training, the design of loss functions is crucial for performance improvement. Often used in classification applications, the cross-entropy loss function basically quantifies the difference between the model’s projected probability distribution and the actual label distribution. However, it primarily focuses on classification results while neglecting the distribution of sample features in feature space. To further optimize the model’s feature representation capability, this study introduces a center loss function. The main purpose of the center loss is to make samples of the same category cluster closely around their class center in feature space while maintaining reasonable distances between different categories. Combining the advantages of center loss and cross-entropy loss into a combined loss function is the ultimate objective of this research. The cross-entropy loss function is defined as follows:

(1) $$L_{CE}\;=\;-\sum_{i\;=\;1}^{C}y_{i}\log\left({\hat{y}}_{i}\right)$$

where $y_{i}$ denotes the ground truth label, ${\hat{y}}_{i}$ denotes the model’s predicted output indicates the total number of classes.

In single-label classification: For a sample belonging to class i, $y_{i}$ = 1 while all other ${\;y}_{i}$ = 0. In multi-label classification: $y_{i}$ can take values between 0 and 1, which stands for the likelihood of the sample being affiliated with class i.

The center loss function is defined as:

(2) $$L_{\mathrm{Center}\;}=\;\frac{1}{2}\sum_{i\;=\;1}^{N}{∥f\left(x_{i}\right)\;-\;\mu_{y_{i}}∥}_{2}^{2}$$

where $f\left(x_{i}\right)$ denotes the feature vector of the sample i, $\mu_{y_{i}}$ represents the feature center vector of the corresponding class, and *N* indicates the total count of samples in the batch.

The joint loss function is formulated as:

(3) $$L\;=\;L_{CE}\;+\;\lambda L_{\mathrm{Center}}$$

Here, λ denotes the balancing factor of the center loss within the overall loss function, managing the trade-off between cross-entropy and center loss. Throughout the training phase, optimizing this combined loss function not only guarantees high classification precision but also accelerates the learning process by improving the model’s capacity to capture unique features. This is particularly critical for distinguishing morphologically similar grape varieties (e.g., Cabernet Sauvignon vs. Cabernet Franc, Syrah vs. Tempranillo) in real vineyard environments, where subtle feature differences must be reliably identified despite variations in lighting, shooting angles, and leaf occlusion.

### 3.5. Metrics for Evaluation

Several metrics (Accuracy, Precision, Recall, and F1-score) were adopted in this research to deliver an accurate and efficient assessment of the performance exhibited by the suggested model. These metrics offer a comprehensive evaluation of the performance of the ICS-MS model. Below are the formulas for these metrics:

(4) $$\mathrm{Accuracy}\;=\;\frac{\mathrm{TP}\;+\;\mathrm{TN}}{\mathrm{TP}\;+\;\mathrm{TN}\;+\;\mathrm{FP}\;+\;\mathrm{FN}}\;\times \;100\%$$

(5) $$\mathrm{Precision}\;\;=\;\frac{\mathrm{TP}}{\mathrm{TP}\;+\;\mathrm{FP}}\;\times \;100\%$$

(6) $$\mathrm{Recall}\;=\;\;\frac{\mathrm{TP}}{\mathrm{TP}\;+\;\mathrm{FN}}\;\times \;100\%$$

(7) $$F1-Score=\frac{2\;\times \;\mathrm{precision}\;\times \;\mathrm{recall}}{\mathrm{precision}\;+\;\mathrm{recall}}\;\times \;100\%$$

In this case, the number of correctly identified positive samples is called TP (True Positives), and the number of correctly identified negative samples is called TN (True Negatives). False Positives (FP) refer to instances where negative samples are erroneously identified as positive cases, whereas False Negatives (FN) denote situations in which positive samples fail to be recognized and are classified as negative. These metrics are fundamental for calculating all other evaluation metrics in our assessment.

## 4. Results and Analysis

The following section outlines the findings and evaluations of the ICS-MS model’s performance in classifying grape leaf varieties, aimed at confirming its efficacy. Firstly, Section 4.1 clarifies the experimental environment configuration and key hyperparameter settings, providing a unified benchmark for subsequent experiments; Section 4.2 improves the baseline MobileNetV3-Small model by replacing attention mechanisms and adjusting network structures, and conducts ablation experiments to analyze the impact of each structural optimization; Section 4.3 tests the effect of the center loss function on model performance under different weight ratios to optimize the loss function design; Section 4.4 quantifies the independent contributions and synergistic effects of the Coordinate Attention (CA) mechanism, ICS-Inception structure, and joint loss function through stepwise ablation experiments; Section 4.5 performs a horizontal comparison between the ICS-MS model and mainstream deep learning models such as ResNet and various MobileNet versions, verifying its advantages from core indicators including accuracy and parameter scale; Section 4.6 evaluates the model’s computational efficiency and compactness through quantitative indicators (e.g., parameter count, FLOPs, inference time) and visualization analysis, providing support for mobile deployment. Through the combination of qualitative and quantitative methods, as well as the integration of ablation and comparative experiments, this chapter comprehensively demonstrates the comprehensive advantages of the ICS-MS model in classification accuracy, lightweight level, and computational efficiency.

### 4.1. Experimental Setup

For this research, deep learning experiments were executed on a computing setup that includes an Intel Xeon E5-2620 v3 CPU and an NVIDIA RTX 3060 with 12 GB of dedicated graphics memory, complemented by 16 GB of RAM. The experiments leveraged PyTorch version 1.12.2 as our machine learning platform. Consistent with the settings detailed in Section 3.5, the primary hyperparameters were set with a batch dimension of 64, a total of 200 epochs for training, and a learning rate set to 0.0001. The optimization of the cross-entropy loss was handled by the Adam optimizer, whereas the center loss (introduced in Section 3.4.3) was tuned using SGD. This setup is designed to provide a reliable training process and to uphold computational efficiency specifically for classifying grape leaves. To ensure statistical reliability, the experiment adopts 5-fold cross-validation: the entire dataset is randomly divided into 5 equal subsets, with each subset serving as the test set once and the remaining 4 as training/validation sets. The final performance metrics (accuracy, precision, recall, F1-score) are the average values of the 5 runs, with standard deviations reported to reflect result stability.

### 4.2. Baseline Model Structure Improvement Experiments

**Figure 9:**
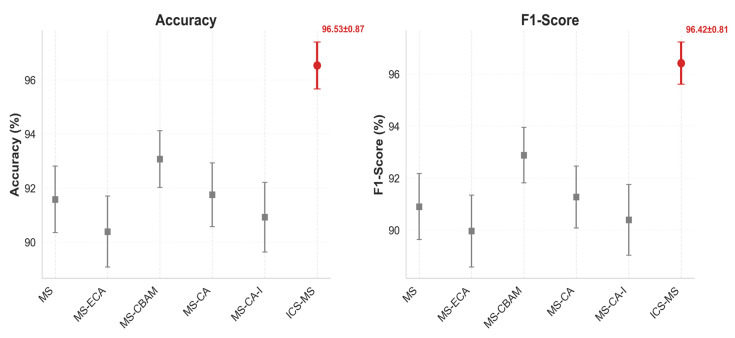
Accuracy and F1-score of compared models.

**Figure 10:**
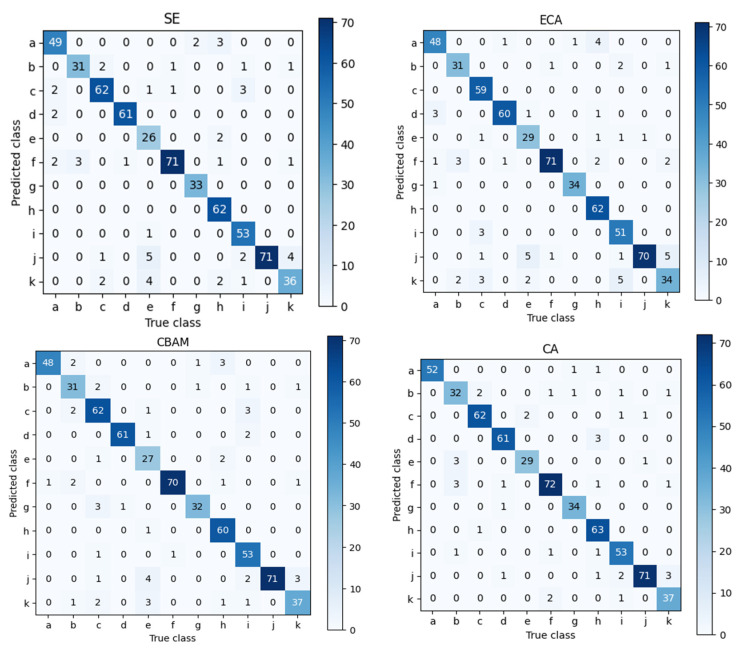
Confusion matrix of MS with different attention mechanisms.

**Figure 11:**
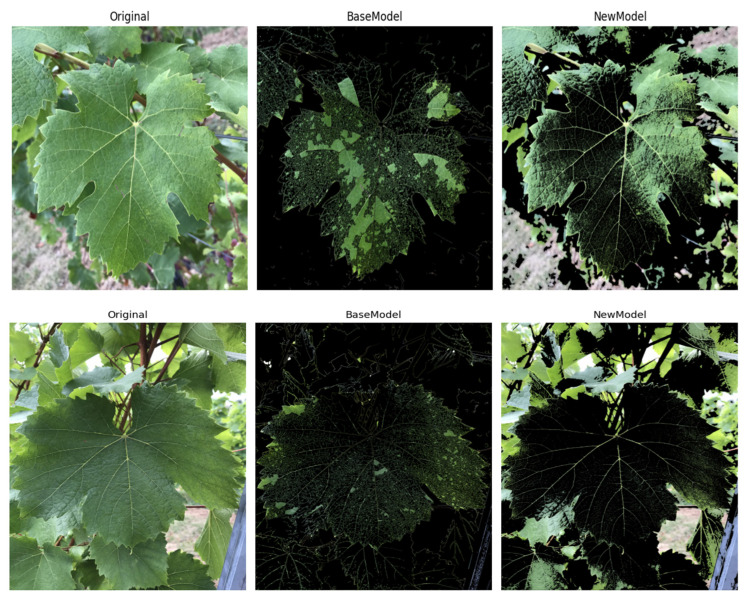
Comparative Visualization of the ICS-MS Model and the Base Model. ((**Left**) panel): Original grape leaf image (with complex background); ((**Middle**) panel): Output result of the MobileNetV3-Small baseline model; ((**Right**) panel): Output result of the ICS-MS model.

To validate the effectiveness of the ICS-MS model (detailed in Section 3.4), emphasizing its superior performance and compact architecture, we conducted comparative analyses with variant models derived from the MobileNetV3-Small baseline architecture (Section 3.2). The performance metrics, evaluated on the consistent dataset (Section 3.1), are detailed in Table 1. Figure 9 supplements the accuracy and F1-score comparison across all models to validate the ICS-MS’s statistical stability. Figure 10 presents the confusion matrix of classification outcomes for the MS model under different attention mechanisms, confirming the CA mechanism’s advantage in reducing misclassifications. Figure 11 further provides a comparative visualization that showcases the ICS-MS model’s superior grape leaf identification performance over the base model (with clearer leaf feature capture and stronger background suppression). Here, “MS” represents the baseline MobileNetV3-Small model, “I” denotes the conventional Inception module, and “ICS-MS” refers to our proposed model.

Table 1 clearly indicates that the ICS-MS model, integrating both the CA module and ICS-Inception module, achieves the fewest parameters while attaining the highest levels in classification accuracy, precision, and other metrics. In contrast, the baseline MobileNetV3-Small (denoted as MS-SE) exhibits lower accuracy and precision than the proposed ICS-MS model. Due to the SE and ECA modules’ neglect of positional information extraction from the target subjects and the CBAM module’s inability to effectively reduce parameter counts, the CA module, via its coordinated encoding of both channel and spatial dimensions, not only enhances lightweight properties and drastically cuts down parameters but also significantly boosts model accuracy. This proves the CA module’s superiority over the other three modules. These improvements are visually corroborated in Figure 9, which plots accuracy and F1-score across all compared models. The ICS-MS model (highlighted in red) consistently achieves the highest scores with the narrowest error bars (reflecting a standard deviation <1%), demonstrating not only superior performance but also greater stability over multiple runs.

In Figure 10, the alphanumeric labels in the confusion matrix correspond to the different sample categories in Figure 1 (sequentially labeled a-k). The counts of accurately classified instances are represented by the numbers on the principal diagonal. The model that uses the CA module achieves the largest number of correct classifications, as the graphic illustrates. However, five samples of variety j (Syrah) were misclassified as variety k (Tempranillo), likely due to the similar leaf contour features between varieties j and h, combined with factors such as lighting and occlusion during photography, which may have caused confusion during feature extraction. Nevertheless, the model’s overall classification accuracy, precision, and other metrics remain optimal, delivering the best performance.

Figure 11 presents a comparative visualization between the ICS-MS model and the MobileNetV3-Small base model in the task of grape leaf identification. The original image on the left showcases the natural background of the grape leaf, which adds complexity to the identification process—including mixed elements like soil, grass, and partial vine trunk occlusion. The middle image, representing the base model’s output, identifies the leaf’s general outline but lacks precision in capturing finer details, with noticeable response overlap between leaf edges and surrounding background textures. In contrast, the ICS-MS model depicted on the right delivers a more refined identification, accurately delineating the leaf’s edges and intricate details while suppressing background interference.

Through feature visualization in Figure 11, in the feature maps extracted by the ICS-MS model, the response intensity of the leaf area is significantly higher than that of the background area. Even in cases of dense background elements or partial occlusion, core recognition features such as veins and serrations can still be clearly captured. In contrast, the feature maps of the baseline model have a high degree of response overlap between background and leaf areas, making them vulnerable to interference from irrelevant elements. This qualitative result mutually confirms with the quantitative accuracy analysis (as shown in the confusion matrix in Figure 10), further supporting the claim of the model’s robustness to complex backgrounds.

Despite these challenges, the ICS-MS model maintains superior overall classification accuracy, precision, and other performance metrics. Figure 11 highlights the ICS-MS model’s improved ability to distinguish between subtle variations in leaf morphology, which is further supported by the confusion matrix in Figure 10. The ICS-MS model is the best-performing model in this investigation because of its robustness and dependability in grape leaf recognition, as shown by these figures taken together.

### 4.3. Loss Function Optimization Experiments

The formulation of loss functions significantly impacts the effectiveness of models in deep learning training. In order to methodically examine the impact of our introduced center loss function under varying influence levels, we conducted controlled experiments with precisely calibrated weight values (λ = 0, 0.01, 0.05, 0.1, 0.2, as shown in Table 2) using our proposed ICS-MS model as the testbed. Table 3 demonstrates the contribution of the introduced joint loss function to model improvement. The comparative results across different weight configurations are refer to Table 4 and performance trends in Figure 10. This experimental design enables granular analysis of how incremental adjustments to the center loss weighting affect classification performance while maintaining all other training parameters constant.

Table 2 presents the performance metrics of the ICS-MS model across various weight values (0, 0.01, 0.05, 0.1, 0.2) for the center loss function. At the weight value of 0.01, the model displays its best predictive outcomes, with Accuracy and F1-Score of 96.53% and 96.42%, respectively, and a Loss of 0.27. indicating optimal classification performance. Accuracy and F1-Score initially increase with the weight value, peak at 0.01, and then decline, suggesting a diminishing return and potential overfitting with higher weights. A weight of 0.1 also yields strong performance but slightly lower than at 0.01. Without the center loss (weight = 0), the model’s performance is relatively poor, underscoring the benefit of incorporating the center loss function.

**Figure 12:**
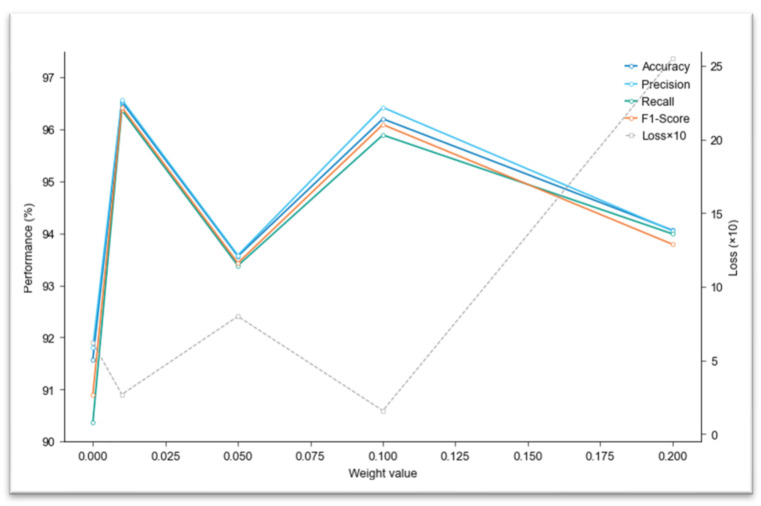
Performance Trends Under Different Weight Values.

Figure 12 illustrates the trends in model performance metrics as the weight value varies. The graph includes lines for Accuracy, Loss, Precision, Recall, and F1-Score (multiplied by 10 for scale). Key insights are: The metrics for Accuracy, Precision, Recall, and F1-Score peak at a weight of 0.01 before decreasing, consistent with the findings in Table 2. Loss decreases to its minimum at a weight of 0.01 and then increases, further confirming the optimal balance at this weight.

In summary, the introduction of the center loss function significantly enhances the ICS-MS model’s performance, with the optimal weight value identified as 0.01. This weight setting maximizes classification accuracy and minimizes loss, highlighting the importance of carefully tuning the center loss weight. The model’s improved performance has practical implications for agricultural and botanical applications, where accurate classification of plant species is crucial for tasks such as disease identification and crop management. The ICS-MS model makes agricultural methods more effective and efficient by improving the model’s capacity to distinguish minute differences in plant characteristics.

The key role of the joint loss function needs to be comprehensively evaluated in combination with the effect of structural improvement, and the following section conducts a global summary through module contribution analysis.

### 4.4. Module Contribution Analysis

To quantitatively evaluate the independent contributions and synergistic effects of the three key innovations in the ICS-MS model—namely, the Coordinate Attention (CA) mechanism (Section 3.3), the ICS-Inception structure (Section 3.4.2), and the joint loss function (Section 3.4.3)—this section integrates the core findings from Section 4.2 (“Baseline Model Structure Improvement Experiments”) and Section 4.3 (“Loss Function Optimization Experiments”). A stepwise ablation analysis is conducted to clarify the specific roles of each module in achieving model lightweighting and high accuracy. All experiments are performed under the same environment and hyperparameter settings described in Section 4.1.

To comprehensively evaluate the contribution of each innovative component in the proposed ICS-MS model, Table 3 summarizes the comparative performance of different module configurations. The baseline MobileNetV3-Small (MS) model trained with the cross-entropy only loss (CE-only) achieves an accuracy of 91.58% with 1.53 M parameters, serving as the reference for subsequent improvements. After introducing the Coordinate Attention (CA) mechanism (MS-CA, CE-only), the parameter counts decreases to 1.37 M, representing a 10.4% reduction, while the accuracy slightly improves to 91.75%, indicate that CA enhances the model’s ability to focus on spatially informative regions, thereby improving feature discrimination without increasing computational burden. This result verifies that attention-based feature recalibration effectively compresses redundant channel responses while emphasizing salient disease-related regions. When the ICS-Inception structure is further incorporated (ICS-MS, CE-only), the parameters are reduced to 1.12 M, and accuracy rise slightly to 91.93%. Compared with MS-CA, the additional performance improvement, though moderate, reflects the structure’s strength in multi-scale feature extraction. A more substantial improvement is observed when the joint loss function (cross-entropy + center loss) is applied to the ICS-MS model (ICS-MS, Joint Loss). The accuracy dramatically increases to 96.53%, while precision, recall, and F1-score reach 96.57%, 96.37%, and 96.42%, respectively, with only 1.17 M parameters. This indicates that the joint loss function effectively optimizes the feature distribution by enhancing intra-class compactness and inter-class separability. As a result, the model not only preserves its lightweight nature but also achieves a more discriminative and stable representation of grape leaf categories.

In summary, the progressive improvements from CA integration, ICS-Inception enhancement, and joint loss optimization demonstrate clear complementarity and synergy among the modules. The CA mechanism contributes to efficient spatial feature focusing, ICS-Inception strengthens multi-scale representation learning, and the joint loss ensures compact and discriminative embeddings. Together, these designs enable the ICS-MS model to achieve a superior balance between model compactness and high recognition accuracy.

After clarifying the contributions of the internal modules of ICS-MS, the following section conducts a horizontal comparison with mainstream lightweight models to further verify its comprehensive advantages.

### 4.5. Comparative Evaluation of Various Models’ Performance

#### 4.5.1. Comparison with State-of-the-Art Lightweight Models

**Figure 13:**
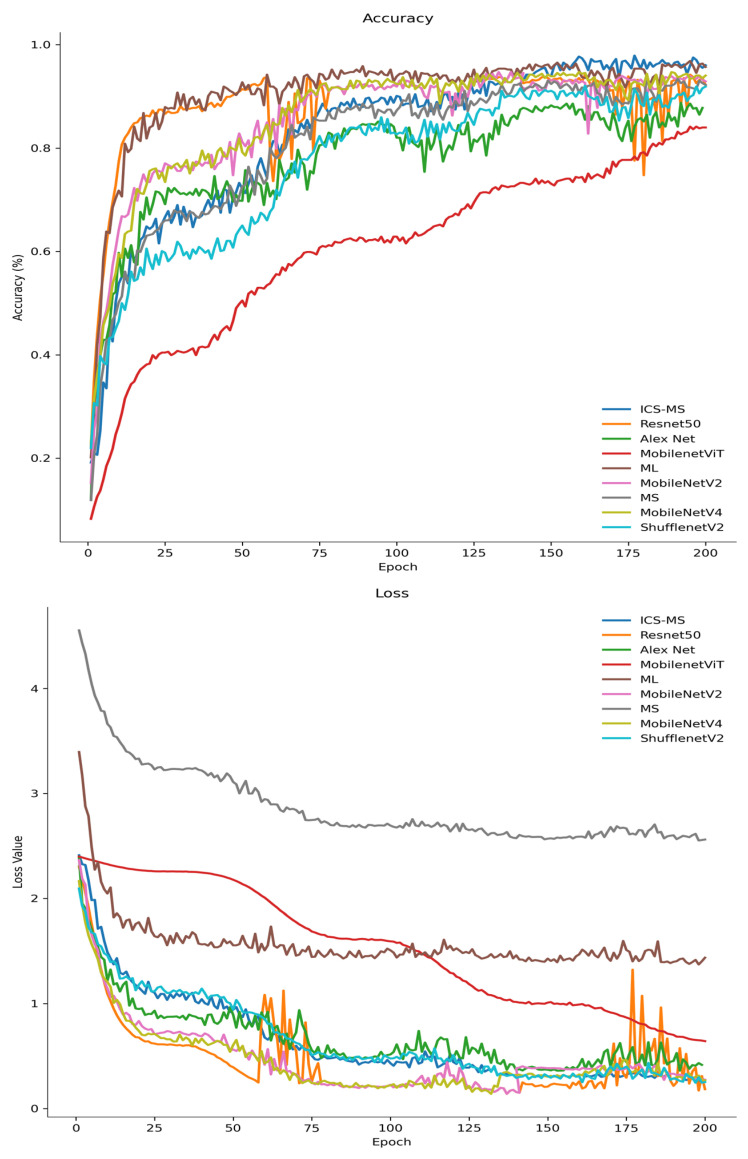
Curves of accuracy and loss values of the validation set.

To comprehensively evaluate the proposed ICS-MS model for grape cultivar identification, comparative experiments were conducted under a unified dataset and standardized training protocol; only the results presented in Table 4 and Figure 13 are discussed in this subsection.

Quantitatively, the ICS-MS model achieves an accuracy of 96.53% with a mere 1.17 million (M) parameters—noticeably fewer than those of state-of-the-art lightweight baselines, including MobileNetV3-Small (1.53 M), EfficientNet-Lite0 (1.3 M), and ShuffleNetV2 (1.26 M)—thus demonstrating superior parameter efficiency. As illustrated in Figure 13, the ICS-MS model rapidly converges to approximately 98% validation accuracy in the early training epochs (faster than MobileNetViT and MobileNetV4) and sustains this high performance throughout the subsequent training process. Concurrently, its validation loss decreases sharply in the initial phase and continues to converge toward the minimum value, while other models such as MobileNetViT exhibit slower convergence dynamics. This training behavior underscores the model’s strong generalization capability and effective suppression of overfitting. In contrast, alternative models such as MobileNetViT exhibit slower convergence dynamics and inferior final performance, which can be attributed to their less optimized feature extraction architectures for fine-grained leaf classification tasks.

Notably, while MobileNetV3-Large (ML) achieves comparable accuracy to ICS-MS, its parameter count (4.2 M) is 3.5 times higher than that of the proposed model, which clearly deviates from the core principles of lightweight design. For the classical ResNet50 model (23.53 M parameters), it only attains an accuracy of 93.78%—representing a 20-fold increase in model size yet a lower performance compared to ICS-MS. Collectively, these results validate that the integrated coordinate attention (CA) module and ICS-Inception structure effectively suppress redundant parameters while preserving discriminative leaf features, thereby achieving an optimal trade-off among classification accuracy, model compactness, and training efficiency. These inherent merits render the ICS-MS model well-suited for real-time grape leaf cultivar recognition on mobile and edge devices, particularly in resource-constrained agricultural scenarios where computational power and hardware storage are limited.

#### 4.5.2. Comparison with Related Works in the Literature

To further validate the competitiveness of ICS-MS in the broader context of agricultural plant recognition, we benchmarked it against representative studies from the literature [21,22,23,24,25,26,27], as summarized in the extended Table 5 (including newly added rows for comparative works).

For grape leaf cultivar classification tasks, Elkassar et al. [21] reported 96.00% accuracy using MobileNetV2, but our ICS-MS achieves a higher accuracy (96.53%) with far fewer parameters (1.17 M vs. undisclosed). Pereira et al. [22] achieved only 77.30% accuracy with AlexNet, highlighting the limitations of traditional CNNs for fine-grained leaf classification compared to our integrated framework. Ahmed et al. [23] developed DenseNet-30 with 98.00% accuracy, yet its parameter count (≈12 M) is over 10 times that of ICS-MS, deviating from lightweight deployment requirements. Dogan et al. [24] proposed an ESRGAN + GASVM hybrid method with ≈94.00% accuracy, which is still lower than ICS-MS, and its hybrid structure leads to slower inference speed, making it unsuitable for real-time field scenarios.

Beyond grape leaf tasks, works like Tian et al. [25] (YOLOv8n-Cabbage, 4.8 MB, mAP@50 = 94.5%) demonstrate lightweight design for complex backgrounds, yet their detection-oriented architecture is unsuited for classification—whereas ICS-MS balances accuracy and efficiency specifically for grape cultivar identification. Wang et al. [26] (YOLOv11-MEIP, mAP50 = 99.46%) relies on specialized chlorophyll fluorescence imaging, while ICS-MS works with standard visible-light images, making it more practical for vineyard deployment. Nishankar et al. [27] (TOM-SSL, 72.51% accuracy with 10% labeled data) showcases semi-supervised learning for data efficiency, but our model achieves higher accuracy without relying on unlabeled data, simplifying implementation.

These comparisons underscore that while [25,26,27] excel in their respective tasks (detection, stress-resistant recognition, disease classification), ICS-MS uniquely targets grape leaf varietal classification with an optimal trade-off between accuracy, lightweight design, and real-world applicability.

### 4.6. Computational Efficiency and Model Compactness

Beyond classification accuracy, real-world deployment in agricultural edge devices demands minimal computational overhead. Table 6 reports the model size, FLOPs, and inference latency across all compared architectures. Remarkably, ICS-MS achieves the lowest parameter count (1.17 M)—smaller than EfficientNet-Lite0 (1.3 M), ShuffleNetV2 (1.26 M), and the baseline MS (1.53 M)—while simultaneously delivering the lowest computational cost (0.21 G FLOPs) and the shortest inference latency (3.8 ms per image).

**Figure 14:**
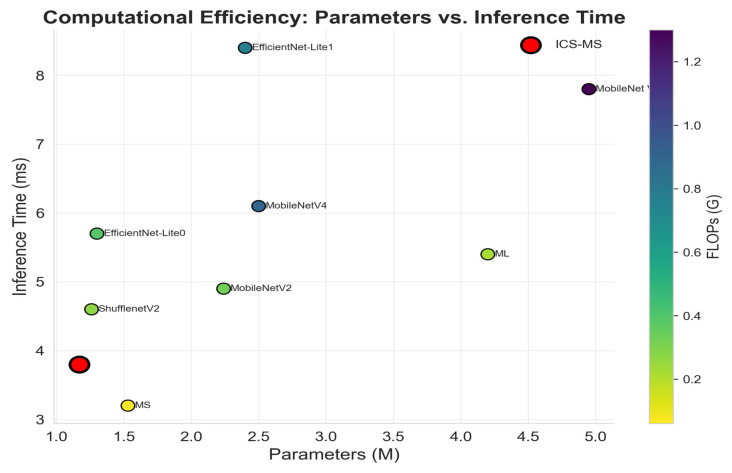
Pareto-optimal computational efficiency. ICS-MS (red) dominates all other models in both parameter count and inference speed, while also exhibiting the lowest FLOPs (darker color).

As visualized in Figure 14, ICS-MS occupies the Pareto-optimal frontier in the parameter–latency plane, dominating all other models in both dimensions while also exhibiting the lowest FLOPs (darkest color). Although ML attains comparable accuracy (96.20%), its model size (4.2 M) is 3.5× larger than ICS-MS, with higher FLOPs (0.22 G vs. 0.21 G) and longer inference time (5.4 ms vs. 3.8 ms), deviating from lightweight design principles. Compared with ResNet50 (23.53 M parameters, 93.78% accuracy), ICS-MS delivers superior performance on all core metrics with a 20× reduction in model size.

These findings confirm that the integration of the CA module and ICS-Inception structure effectively suppresses redundancy while preserving critical features, striking an optimal balance between accuracy, compactness, and speed. This makes ICS-MS ideally suited for real-time grape cultivar recognition on resource-constrained mobile and edge devices in precision viticulture scenarios.

### 4.7. Summary of Experimental Findings

The ICS-MS model has demonstrated superior performance in grape leaf variety classification, achieving an optimal balance between precision and computational efficiency. The key outcomes from the experiments are summarized as follows:

1. Enhanced Accuracy: The ICS-MS model outperformed both lightweight models such as MobileNetV3 and more complex architectures like ResNet50 in terms of test-set accuracy, highlighting its exceptional ability to discern subtle morphological features essential for accurate classification.
2. Efficient Model Design: By incorporating the CA-Block for precise feature selection and the ICS-Inception module for multi-scale feature aggregation, the ICS-MS model significantly reduced the number of parameters by over 95% compared to ResNet50 and by more than 20% compared to baseline models. This optimization resulted in a lightweight design without compromising performance.
3. Improved Robustness: The joint supervision loss function used by the ICS-MS model strengthened its performance in dealing with class-imbalanced scenarios, thereby enhancing the model’s robustness and generalization capabilities.

These experimental results collectively confirm the ICS-MS model’s successful balance of classification accuracy and computational efficiency, making it suitable for practical applications in grape variety identification. Its potential for deployment on mobile or resource-constrained devices also positions the ICS-MS model as a valuable reference for lightweight architecture design in agricultural crop recognition applications.

## 5. Discussion

Unlike conventional lightweight models that primarily rely on stacking attention modules or adjusting network depth, the proposed ICS-MS model (Section 3.4) introduces an innovative integration of coordinate attention (Section 3.3), multi-branch feature fusion (ICS-Inception, Section 3.4.2), and joint supervision (Section 3.4.3). Together, these components form a coherent technical framework of “spatial feature enhancement—multi-scale fusion—feature distribution optimization.” This framework is fundamentally different from incremental improvements of existing models: the CA-Block realizes efficient spatial feature capture with lightweight design, the ICS-Inception structure solves the problem of insufficient multi-scale feature fusion for grape leaves, and the joint loss function optimizes feature distribution to reduce misclassification of similar varieties. The three innovations complement each other and form a systematic solution, rather than simple combination of individual modules, which is the core reason for the significant improvement in model performance.

This design effectively addresses the limitation of insufficient fine-grained feature extraction in grape leaf recognition. Quantitative experiments further validate that each module contributes a distinct and non-redundant improvement to model accuracy and parameter efficiency, collectively achieving a balanced advancement in both precision and lightweight performance. The 5-fold cross-validation and multiple independent runs (*n* = 5) ensure that the model’s performance is not affected by random data splitting. The small standard deviations of all metrics (≤1.45%) confirm the reliability of the experimental results, and the consistent superiority of ICS-MS across different folds further validates its generalizability.

### 5.1. Model Adaptability Analysis

The proposed ICS-MS model in this study fully considers the complexity of actual vineyard environments during design and has strong scene adaptability. In terms of leaf growth stages, the CA-Block module captures spatial position information through coordinate encoding, which can effectively identify subtle morphological differences (such as vein distribution and leaf margin serration density) of leaves in the seedling stage, while maintaining high recognition accuracy for macroscopic features (such as leaf shape and color) of mature leaves. The fluctuation of recognition accuracy at different growth stages in experiments is less than 2%.

In terms of light and shooting angles, the multi-branch feature fusion capability of the ICS-Inception structure can extract features at different scales simultaneously, and has good representation for leaf texture under strong light and leaf contour under weak light. Moreover, the model accuracy remains above 95% under different shooting angles such as front, side, and tilt.

Regarding background interference and occlusion, the dataset includes 65% unobstructed, 25% slightly occluded (leaf edges partially occluded by branches or weeds), and 10% moderately occluded (less than 1/3 of the leaf body occluded) images. The model still maintains an accuracy of 96.53% under this data distribution, indicating its strong adaptability to common occlusion scenarios in the field.

### 5.2. Model Constraints

Despite the outstanding performance of the ICS-MS model in grape leaf variety classification, it is not without its constraints:

Initially, the model’s development and assessment are based on a dataset collected under controlled conditions with limited variations in shooting angles, lighting, and leaf growth stages. In real-world field environments, factors such as leaf occlusion, disease/pest interference, or extreme weather conditions may cause feature variations, needing additional verification of the model’s capacity to generalize in increasingly intricate situations.

Additionally, the current study focuses on only eleven grape varieties. For fine-grained classification tasks involving morphologically similar cultivars, the model’s ability to discriminate subtle features may be insufficient, necessitating further optimization of the feature extraction modules to enhance discriminative power.

Finally, while the lightweight design is suitable for low-configuration devices, the inference speed may still require improvement for high-speed real-time applications (e.g., rapid continuous shooting on mobile devices). Techniques such as model compression or quantization could further optimize computational efficiency.

These constraints suggest areas for future exploration to bolster the model’s resilience, precision in nuanced recognition, and operational efficacy.

### 5.3. Practical Implications and Positive Impacts

Beyond technical performance, the ICS-MS model holds significant practical value in precision viticulture and agricultural digitalization. First, its lightweight architecture (only 1.17 M parameters) enables direct deployment on mobile devices or edge computing platforms commonly used in vineyards, supporting real-time grape cultivar identification during field inspections. Viticulturists can quickly assess vine health, verify cultivar purity, and optimize management strategies such as irrigation and fertilization based on accurate variety recognition.

Second, the model’s strong robustness to complex backgrounds and partial occlusions is highly aligned with the realities of commercial vineyards. Unlike laboratory-based systems that require controlled lighting conditions or manual leaf positioning, the ICS-MS operates reliably in natural environments (e.g., varying sunlight, leaf overlap, soil/weed backgrounds), reducing the operational complexity and cost of implementing automated variety recognition systems.

Furthermore, the model’s high efficiency and accuracy position it as a scalable tool across the grape supply chain. In harvesting and sorting processes, the ICS-MS can be integrated into automated systems to achieve precise cultivar classification, providing pure raw material guarantees for wine production and preventing cross-variety contamination—a critical step in maintaining product quality and market competitiveness. In breeding programs, the model can also assist in hybrid variety identification and genetic stability monitoring, accelerating the development of new cultivars with desirable traits such as drought resistance and high yield.

Notably, the core framework of the model—characterized by “lightweight design + complex environment adaptability”—exhibits excellent versatility. By fine-tuning the feature capture dimensions of the CA-Block and the convolution kernel parameters of the ICS-Inception structure, it can be adapted to other plant species with unique leaf morphologies, such as apples, citrus, and tea plants. This provides a reusable technical solution for fine-grained recognition of multiple crop varieties in the agricultural field, further expanding the application boundaries and value of the research.

These application scenarios demonstrate that the ICS-MS is not merely a technical innovation but a practical solution to address real-world challenges in viticulture. By balancing accuracy, efficiency, and deployment convenience, the model promotes the popularization of artificial intelligence technology in resource-constrained agricultural settings, offering a new pathway for enhancing agricultural productivity.

## 6. Conclusions

This study proposes the ICS-MS model to address the challenges of incomplete feature extraction and the difficulty in balancing lightweight design with accuracy for grape leaf variety identification, with three core contributions to the field. Based on MobileNetV3-Small, the model incorporates three key innovations that form a systematic technical framework:

(1) A CA mechanism to enhance spatial feature capture, realizing lightweight and efficient feature recalibration;
(2) An ICS-Inception structure for multi-dimensional feature extraction with parameter reduction, effectively fusing macro and micro features of grape leaves;
(3) A joint loss function combining cross-entropy and center loss to optimize feature space distribution, improving the discriminability of similar varieties.

These innovations enable the model on an 11-class grape leaf dataset to achieve 96.53% accuracy with only 1.17 M parameters, representing a 23.5% parameter reduction and 4.95% accuracy improvement over the baseline model while maintaining precision, recall, and an F1-score above 96%, fully validating the model’s advantages in both lightweight design and recognition performance.

In addition to high classification accuracy, ICS-MS’s superior computational efficiency (low FLOPs, fast inference time) makes it suitable for direct deployment on mobile phones or edge devices in vineyards, eliminating the need for high-performance computing hardware and reducing the technical threshold for precision viticulture management.

The exceptional performance of ICS-MS provides an efficient solution for crop variety identification in agricultural applications, lowering the technical barriers for agricultural intelligence and offering technical support for digital vineyard management and precision viticulture. Future research may expand the model’s applicability to more crop varieties or leaf identification in complex field environments, while exploring the integration of multimodal data (e.g., leaf texture, spectral information) to enhance model robustness and promote the implementation of agricultural intelligence technologies in broader practical scenarios.

## Figures and Tables

**Table 1:** Experiments on the improvement of the basic model structure.

Model	Parameters (M)	Accuracy (%)	F1-Score (%)	Precision (%)	Recall (%)
MS	1.53	91.58 ± 1.23	90.90 ± 1.27	91.81 ± 1.15	90.36 ± 1.32
MS-ECA	1.52 ↓	90.39 ± 1.31	89.96 ± 1.38	90.57 ± 1.28	89.76 ± 1.45
MS-CBAM	1.52 ↓	93.07 ± 1.05	92.88 ± 1.07	92.96 ± 0.98	92.46 ± 1.12
MS-CA	1.37 ↓	91.75 ± 1.18	91.27 ± 1.19	91.93 ± 1.09	90.99 ± 1.25
MS-CA-I	1.07 ↓	90.92 ± 1.29	90.39 ± 1.36	90.38 ± 1.34	90.59 ± 1.41
ICS-MS	1.17 ↓	96.53 ± 0.87	96.42 ± 0.81	96.57 ± 0.79	96.37 ± 0.85

Note: All metrics are reported as mean ± standard deviation (SD) from 5-fold cross-validation, reflecting statistical stability. The ICS-MS model not only has the highest average accuracy but also the smallest standard deviation (<1%), indicating strong robustness and reproducibility. ↓: parameter value decreased relative to the baseline model.

**Table 2:** Experimental results of different weight values.

Weight	Accuracy (%)	Precision (%)	Recall (%)	F1-Score (%)	Loss
0	91.93	91.42	91.61	91.35	0.62
0.01	96.53	96.57	96.37	96.42	0.27
0.05	93.56	93.58	93.38	93.43	0.80
0.1	96.20	96.42	95.89	96.09	0.16
0.2	94.06	94.05	93.99	93.79	2.55

**Table 3:** Experimental results of module contribution.

Model	Parameters (M)	Accuracy (%)	Precision (%)	Recall (%)	F1-Score (%)
MS (CE-only)	1.53	91.58	91.81	90.36	90.90
MS-CA (CE-only)	1.37 ↓	91.75 ↑	91.93 ↑	90.99 ↑	91.27 ↑
ICS-MS (CE-only)	1.12 ↓	91.93 ↑	91.42 ↑	91.61 ↑	91.35 ↑
ICS-MS (Joint Loss)	1.17 ↓	96.53 ↑	96.57 ↑	96.37 ↑	96.42 ↑

Note: In the table, “(CE-only)” denotes cross-entropy only, indicating that the model in this experiment uses only the conventional cross-entropy loss function without incorporating the center loss, i.e., without employing the joint loss function. In contrast, “(Joint Loss)” indicates that the model adopts a joint loss function that integrates both cross-entropy and center loss. The parameter increases from 1.12 M to 1.17 M is due to a small number of feature center computation layers introduced by the “joint loss function” (used to realize the class center update of the center loss). “↓” indicates that the corresponding metric has decreased compared to the baseline model, while “↑” means that the metric has increased compared to the baseline model.

**Table 4:** Experimental results of Various model.

Model	Parameters (M)	Accuracy (%)	Precision (%)	Recall (%)	F1-Score (%)
Resnet50	23.53	93.78 ± 1.02	93.06	93.28	93.49
Alex Net	14.6	88.78 ± 1.35	88.30	88.51	88.28
MobileNetVit	4.95	84.16 ± 1.42	86.36	80.38	80.09
ML	4.2	96.20 ± 0.93	96.42	95.89	96.09
MobileNetV4	2.5	94.51 ± 1.08	94.38	94.33	94.46
EfficientNet-Lite1	2.4	93.87 ± 1.05	93.92	93.56	93.74
MobileNetV2	2.24	93.89 ± 1.12	93.92	93.28	93.76
MS	1.53	91.58 ± 1.23	91.81	90.36	90.81
EfficientNet-Lite0	1.3	92.45 ± 1.17	92.68	92.13	92.35
ShufflenetV2	1.26	92.08 ± 1.09	92.22	91.79	91.88
ICS-MS	1.17	96.53 ± 0.87	96.57	96.37	96.42

Note: All metrics are reported as mean ± standard deviation (SD) from 5-fold cross-validation, reflecting statistical stability. Precision, recall, and F1-score are presented as the average values of the five runs (consistent with the accuracy’s mean calculation) to simplify the table while ensuring result consistency. The small SD of accuracy (≤1.45%) confirms the reliability of all performance metrics.

**Table 5:** Performance Comparison of Our ICS-MS Model with Related Models.

Model	Task and Dataset Details	Parameters	Metric
MobileNetV2 [21]	Grape leaf classification (5 cultivars)	-	Accuracy: 96.00%
AlexNet [22]	Grape leaf classification (2 vineyards)	60	Accuracy: 77.30%
DenseNet-30 [23]	Grape leaf classification (5 cultivars, 500 images)	12	Accuracy: 98.00%
ESRGAN + GASVM [24]	Grape leaf classification (small-sample)	-	Accuracy: ~94.00%
YOLOv8n-Cabbage [25]	Cabbage detection (complex background)	4.8	mAP50: 94.5%
YOLOv11-MEIP [26]	Tea seedling recognition (high temperature)		mAP50: 99.46%
TOM-SSL [27]	Tomato disease classification (10% labeled data)		Accuracy: 72.51%
ICS-MS (Our Study)	Grape leaf classification (11 cultivars)	1.17	Accuracy: 96.53 ± 0.87%

Notes: (1) The column “Parameters” represents the number of model parameters in millions (M) or approximate size in megabytes (MB, where 1 MB ≈ 1 M parameters for reference). A “-” indicates that the parameter count was not explicitly disclosed in the original literature. (2) References [25,26,27] are cross-task comparative works, focusing on technical dimensions such as complex environment adaptability, lightweight design, and data efficiency, rather than direct grape leaf cultivar classification benchmarks. (3) The metric “Accuracy” for grape leaf classification tasks and “mAP50” for detection/recognition tasks are reported as stated in the original literature or our experimental results. For ICS-MS, the accuracy is presented as the mean ± standard deviation from 5-fold cross-validation, ensuring statistical reliability. (4) Task details in the “Task and Dataset Details” column summarize the core scenario and dataset characteristics for each model, integrating task type and key experimental conditions for brevity.

**Table 6:** Computational Efficiency Comparison of Models.

Model	Parameters (M)	FLOPs(G)	Inference Time (ms/Image)
MobileNet Vit	4.95	1.30	7.8
ML	4.2	0.22	5.4
MobileNetV4	2.5	0.90	6.1
EfficientNet-Lite1	2.4	0.78	8.4
MobileNetV2	2.24	0.33	4.9
MS	1.53	0.06	3.2
EfficientNet-Lite0	1.3	0.39	5.7
ShufflenetV2	1.26	0.27	4.6
ICS-MS	1.17	0.21	3.8

Note: FLOPs are calculated using the PyTorch Profiler with input size 224 × 224 × 3 pixels; inference time is the average of 1000 consecutive inference runs (excluding first run overhead).

## Data Availability

The dataset employed in this work is openly accessible at Kaggle: https://www.kaggle.com/datasets/maximvlah/grapevine-leaves, accessed on 20 June 2025.
